# Early Host Cell Targets of *Yersinia pestis* during Primary Pneumonic Plague

**DOI:** 10.1371/journal.ppat.1003679

**Published:** 2013-10-03

**Authors:** Roger D. Pechous, Vijay Sivaraman, Paul A. Price, Nikolas M. Stasulli, William E. Goldman

**Affiliations:** Department of Microbiology and Immunology, University of North Carolina at Chapel Hill, Chapel Hill, North Carolina, United States of America; Yale University, United States of America

## Abstract

Inhalation of *Yersinia pestis* causes primary pneumonic plague, a highly lethal syndrome with mortality rates approaching 100%. Pneumonic plague progression is biphasic, with an initial pre-inflammatory phase facilitating bacterial growth in the absence of host inflammation, followed by a pro-inflammatory phase marked by extensive neutrophil influx, an inflammatory cytokine storm, and severe tissue destruction. Using a FRET-based probe to quantitate injection of effector proteins by the *Y. pestis* type III secretion system, we show that these bacteria target alveolar macrophages early during infection of mice, followed by a switch in host cell preference to neutrophils. We also demonstrate that neutrophil influx is unable to limit bacterial growth in the lung and is ultimately responsible for the severe inflammation during the lethal pro-inflammatory phase.

## Author Summary

Inhalation of the bacterium *Yersinia pestis* results in primary pneumonic plague, a severe necrotizing pneumonia with mortality rates approaching 100% in the absence of timely antibiotic administration. Despite the notoriety of *Y. pestis* as a potential biological weapon and its well-established pandemic potential, very little is known regarding early host-pathogen interactions that lead to the progression of pulmonary infection. *Y. pestis* harbors a type III secretion system (T3SS) for delivery of *Yersinia* outer protein (Yop) effectors into host cells, an early and essential step in pathogenesis. In the work presented here, we identify the host cell targets of *Y. pestis* Yop secretion in the lung. We show that *Y. pestis* initially targets alveolar macrophages, followed by a shift in host cell preference to neutrophils. Through cellular depletion studies, we demonstrate that *Y. pestis* is highly resistant to macrophage- and neutrophil-mediated clearance, and that the accumulation of neutrophils in the lung is responsible for the severe necrotizing pneumonia that develops during the pro-inflammatory phase of pneumonic plague.

## Introduction

The historical impact of *Yersinia pestis* on humanity cannot be understated, as three major pandemics including the “Black Death” of the Middle Ages have been attributed to *Y. pestis* [1], [2]. The bacterium can be transmitted person-to-person via respiratory droplets resulting in primary pneumonic plague, which is the deadliest manifestation of *Y. pestis* infection [1], [2], [3]. In the event of respiratory exposure in humans, mortality rates are nearly 100% with a time to death of typically between four and seven days [1]. Its extreme lethality and history of weaponization have led to the assignment of *Y. pestis* as a Tier 1 Select Agent and compound fears of its intentional release as a weapon of bioterrorism [1].

Using a murine intranasal infection model, our laboratory demonstrated that the primary pneumonic plague syndrome progresses in two distinct phases [4]: an initial “pre-inflammatory phase” characterized by rapid bacterial replication in the lung in the absence of host innate immune responses, followed by a “pro-inflammatory” phase marked by extensive neutrophil influx, a massive pro-inflammatory cytokine storm, and considerable tissue destruction within the lung. In mice and humans, progression into this pro-inflammatory phase invariably results in death without immediate treatment [5], [6]. Recently, our laboratory showed that *Y. pestis* creates a unique protective environment in the lungs of mice that allows for the growth of typically avirulent organisms [7], suggesting that *Y. pestis* suppresses host innate immune responses in the lung early during infection. The mechanism and host cell types involved in this phenomenon remain unknown.

*Y. pestis* utilizes a plasmid-encoded type III secretion system (T3SS) to deliver *Yersinia* effector proteins (Yops) directly to the cytosol of target cells. Injection of Yop effectors is essential for *Y. pestis* virulence and is known to have anti-inflammatory and anti-phagocytic effects on mammalian cells [2], [8], [9]. The pulmonary cells targeted by *Y. pestis* during primary pneumonic plague have yet to be identified. In the work presented here, we use fully virulent *Y. pestis* CO92 expressing a YopE-TEM β-lactamase hybrid protein to identify the host cells targeted in the lung, and to evaluate the effect of depletion of these cell types on the progression of pneumonic plague. We show that the *Y. pestis* T3SS primarily targets macrophages and neutrophils early during the pre-inflammatory phase of disease. We also monitor the host cell dynamics in the lung in response to *Y. pestis* challenge and show that neutrophils are ultimately responsible for the severe necrotizing pneumonia during the pro-inflammatory disease phase. This work is the first identification/evaluation of the host cells targeted by fully virulent *Y. pestis* in the lung, and the results give insight into the dynamic events occurring shortly after pulmonary exposure to the highly lethal pathogen *Y. pestis*.

## Results

### Injection by the T3SS is detectable *in vivo* during primary pneumonic plague

*Y. pestis* injection of Yop effector proteins is essential for virulence during plague. We sought to evaluate *in vivo* injection of Yop effector proteins in the lung using a murine intranasal model of *Y. pestis* infection. Chimeric Yop-TEM β-lactamase proteins have been used previously to demonstrate Yop translocation into target cells of the spleen and lymph nodes *in vivo* and tissue culture macrophages *in vitro* [10], [11], [12]. Briefly, host cells or tissues are infected with *Y. pestis* harboring a Yop-TEM translational fusion, followed by staining of cells with the fluorescent substrate CCF2-AM (Invitrogen). CCF2-AM is a cephalosporin conjugated to the fluorophores coumarin and fluorescein that exhibit fluorescence resonance energy transfer (FRET). Uninfected mammalian cells harboring CCF2-AM can be detected as green fluorescence emission (520 nm); cells that have been targeted for Yop translocation will fluoresce blue (447 nm) due to cleavage of cytosolic CCF2-AM (and disruption of FRET) by the β-lactamase moiety of Yop-TEM. We constructed a strain of *Y. pestis* CO92 (*Y. pestis* YopE-TEM) that expresses both wild-type YopE and the N-terminal 100 amino acids of YopE fused to TEM β-lactamase. YopE is a GTPase activating protein that is highly secreted *in vitro* and is known to inhibit phagocytosis through the disruption of actin microfilaments [8], [9]. Incorporation of the YopE-TEM β-lactamase fusion had no effect on the virulence of *Y. pestis* CO92 as determined by bacterial burden, cytokine, and histopathological analysis.

**Figure 1:**
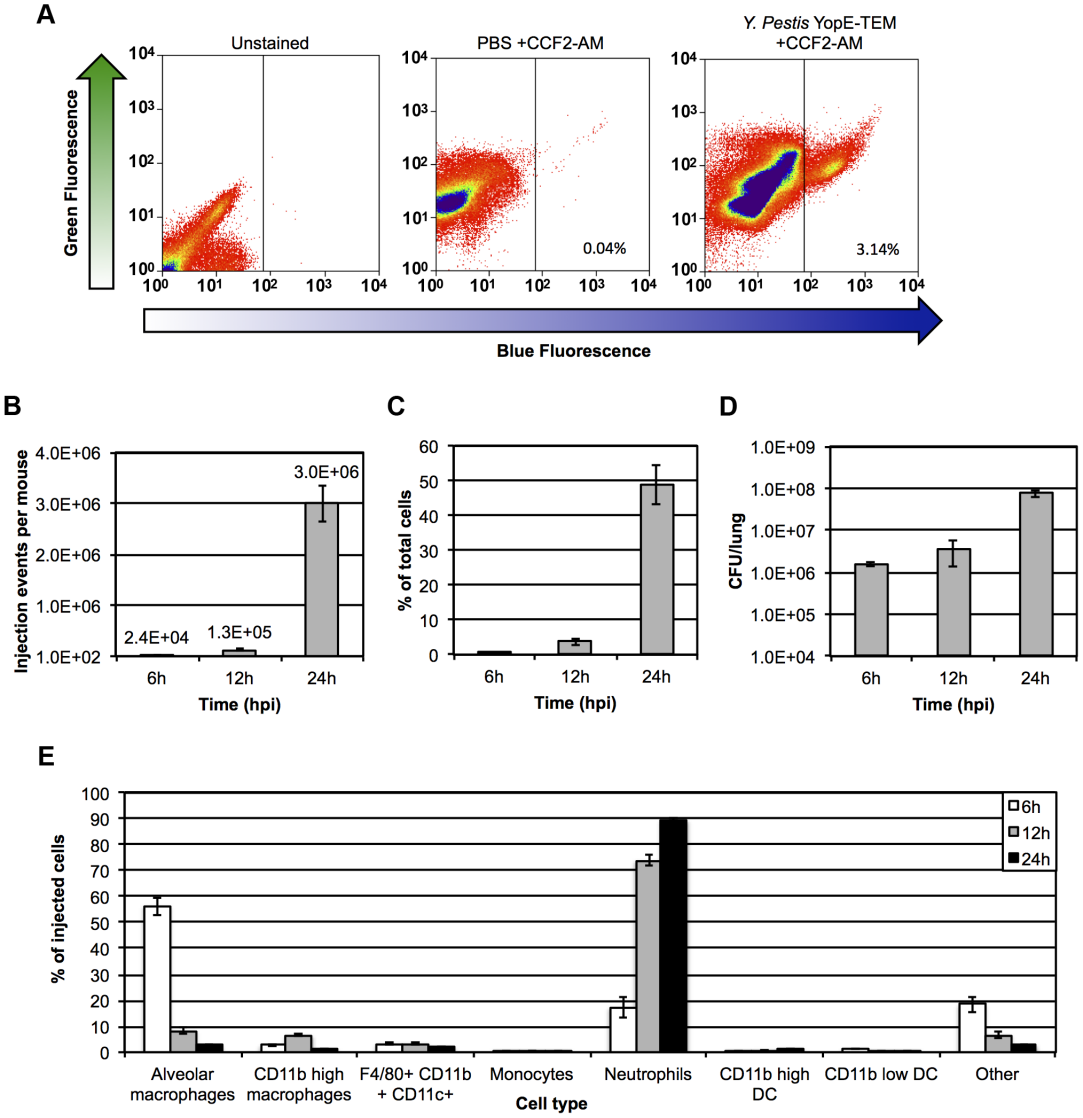
YopE-TEM translocation in the lung during pneumonic plague. (A) Groups of mice were inoculated intranasally with 10^6^ CFU of *Y. pestis* expressing YopE-TEM or PBS alone (mock), followed by staining with CCF2-AM. Lungs were harvested at 12 hpi, and live cells were gated to evaluate blue/green fluorescence by flow cytometry. Cells showing blue fluorescence have been injected with YopE-TEM. Histograms show representative data from a single mouse; (B) Total injection events (cells exhibiting blue fluorescence) in the lungs of mice inoculated with 10^6^ CFU *Y. pestis* YopE-TEM; (C) The percentage of total cells in the lung injected with YopE-TEM was evaluated for samples shown in (B) at 6, 12, and 24 hpi; (D) Lung bacterial burden was evaluated in mice inoculated with 10^6^ CFU *Y. pestis* YopE-TEM. (E) Live injected cells in lungs of mice inoculated with 10^6^ CFU *Y. pestis* YopE-TEM were gated for identification of alveolar macrophages (F480^+^CD11b^low/mid^CD11c^high^), interstitial macrophages (F480^+^CD11b^high^CD11c^mid/low^), F480^+^CD11b^high^CD11c^high^ macrophages, monocytes (F480^−^CD11b^high^Ly-6G^−^), neutrophils (F480^−^CD11b^high^Ly-6G^+^), and CD11b high and low dendritic cells (F480^−^ CD11c^+^); All data are representative of at least three independent experiments with three to five mice at each time point. Error bars represent SEM.

To evaluate YopE-TEM injection *in vivo*, C57BL/6J mice were inoculated with 10^6^ CFU of *Y. pestis* YopE-TEM, and single-cell suspensions were generated from infected lungs at 12 hpi for evaluation by flow cytometry [13]. Lung suspensions stained with CCF2-AM uniformly exhibited green fluorescence compared to unstained samples (Figure 1A). In contrast to mice inoculated with PBS alone, mice infected with *Y. pestis* YopE-TEM exhibited blue fluorescence in a subset of cells (3% of total cells), indicating that YopE-TEM-mediated cleavage of CCF2-AM was detectable *in vivo* by flow cytometry (Figure 1A). For a more detailed analysis, groups of mice were sacrificed at 6, 12, and 24 hpi for flow cytometry evaluation of blue fluorescence. At 6 hpi, roughly 2×10^4^ injection events per mouse were detected, representing approximately 1% of total recovered cells (Figure 1B,C). This number increased over time, reaching roughly 3×10^6^, or 48% of total recovered cells by 24 hpi (Figure 1B,C). The approximately 100-fold increase in injection events coincided with a nearly 100-fold increase in bacterial burden (Figure 1D). These data demonstrate that *Y. pestis* secretion of effectors occurs early during pneumonic plague, and is detectable in the lung using flow cytometry.

### *Y. pestis* targets alveolar macrophages and neutrophils early during primary pneumonic plague

The ability of *Y. pestis* to create a localized protective environment in the lungs early during pneumonic plague suggests that it deactivates host innate immune responses [7]. We hypothesized that *Y. pestis* targets innate immune cell populations early in the lung via type III secretion of Yop effectors. In order to examine the host cell types intoxicated early during infection, mice were sacrificed at 6, 12, and 24 hpi, and whole lung cell suspensions were incubated with CCF2-AM as well as fluorescently labeled anti-CD45 and anti-CD3 antibodies. Cells exhibiting blue fluorescence were gated and analyzed to distinguish between lymphocyte (CD45^+^CD3^+^), leukocyte (CD45^+^CD3^−^), and epithelial/endothelial (CD45^−^CD3^−^) cell populations. At each of the time points examined, >96% of injection events occurred in a population of CD45^+^CD3^−^ cells, indicating that innate immune leukocyte populations were the primary targets for *Y. pestis* translocation of YopE-TEM (Figure S1B). By 24 hpi roughly 40% of CD45^+^CD3^−^ cells were injected with YopE-TEM, whereas less than 3% of the other two populations were injected (Figure S1C).

To identify the leukocyte cell populations targeted by *Y. pestis*, lungs of mice infected with *Y. pestis* YopE-TEM were incubated with CCF2-AM as well as a panel of fluorescently labeled antibodies to cell type-specific markers. Viable cells exhibiting blue fluorescence were gated and analyzed based on fluorescence staining patterns to identify alveolar macrophages, CD11b^high^ interstitial/exudate macrophages, F4/80^+^CD11b^high^ CD11c^high^ macrophages, monocytes, CD11b^high^ and CD11b^low^ dendritic cells (DCs), and neutrophils (Figure S2A). At 6 hpi, alveolar macrophages represented roughly 55% of injected cells (Figure 1E). Neutrophils and cells that are unidentifiable based on our antibody panel (“other”) were the next most prominent target populations, each representing roughly 18% of injected cells. At 12 hpi a dramatic switch in host cell preference occurred, where greater than 70% of injected cells were neutrophils and alveolar macrophages represented less than 10% of injected cells (Figure 1E). By 24 hpi approximately 90% of injected cells were neutrophils, and more than 60% of all recovered neutrophils were positive for YopE-TEM translocation (Figure S2B). In summary, *Y. pestis* initially injects effector Yops into resident alveolar macrophages during pneumonic plague, followed by a shift in host cell preference to neutrophils.

### The shift in *Y. pestis* host cell preference corresponds with an influx of neutrophils into the lung

**Figure 2:**
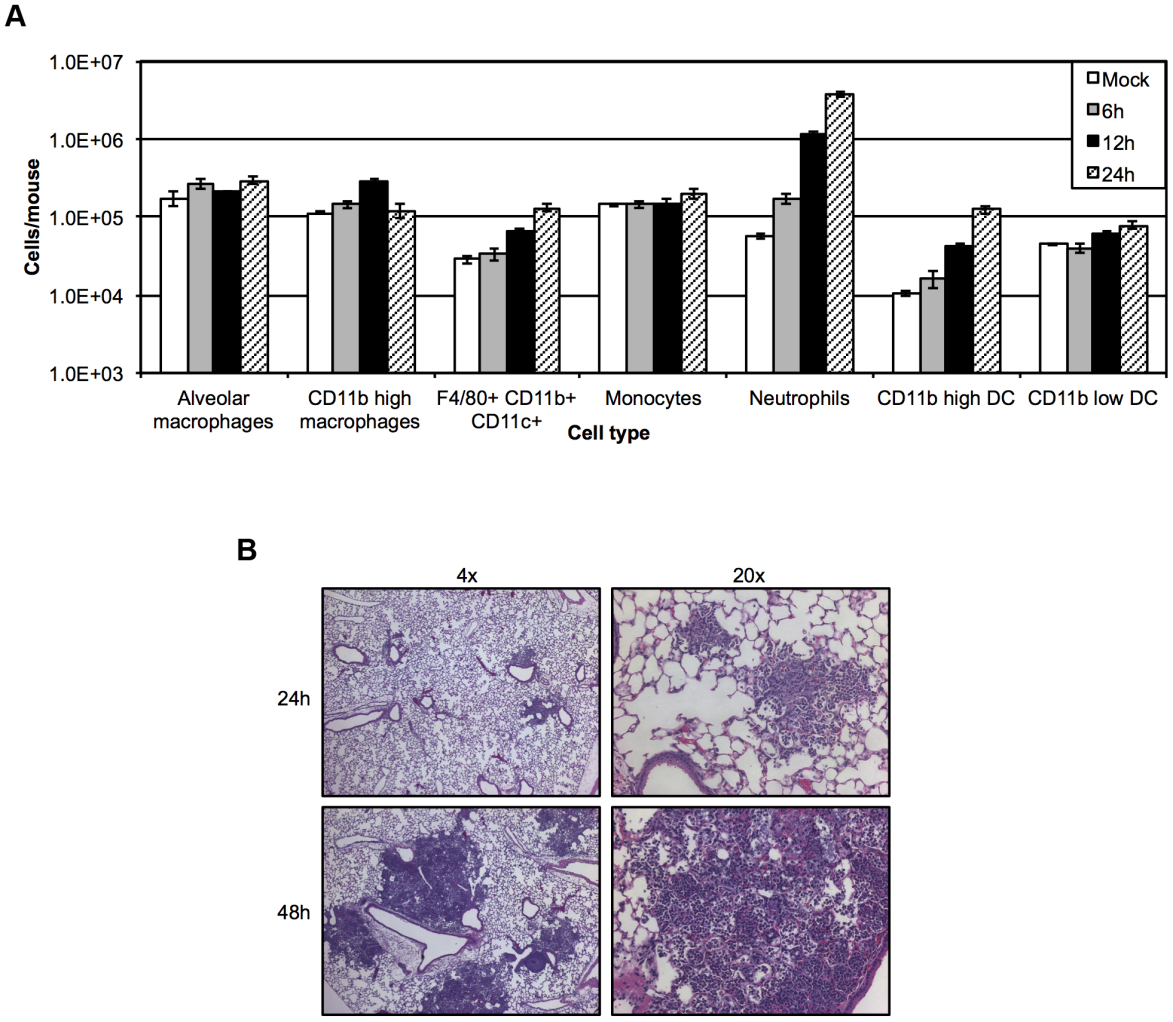
Lung host cell repertoire of mice inoculated intranasally with *Y. pestis*. (A) Total cell numbers of innate immune cell populations in the lungs of mice inoculated with 10^6^ CFU *Y. pestis* YopE-TEM or PBS (mock) at 6, 12, and 24 hpi. (B) *Y. pestis* causes severe inflammation in the lungs that is visible by 48 hpi. Mice were inoculated with 10^6^ CFU *Y. pestis* and lungs were inflated with 10% formalin at 24 and 48 hpi prior to H and E staining. Images are representative of experiments done a minimum of three times. Data are representative of at least three independent experiments with five mice at each time point. Error bars represent SEM.

Understanding how the preferred cellular targets of *Y. pestis* change over time also demands that we assess the host cell repertoire within the infected lung. Flow cytometry analysis revealed increased numbers of neutrophils as early as 6 hpi that reached roughly 50 times those of mock-infected mice by 24 hpi (Figure 2A). Infected mice also showed elevated levels of CD11b^high^ DCs as well as F4/80^+^CD11b^high^CD11c^high^ cells that continued to increase throughout the duration of the pre-inflammatory phase of disease (Figure 2A). Histological analysis of infected lungs showed that the influx of neutrophils resulted in minimal changes in lung pathology at 24 hpi, and small, localized areas of inflammatory infiltrate were found sporadically throughout infected lung sections (Figure 2B). These animals displayed no outward symptoms of disease at this time point. This is in contrast to what was seen at 48 hpi, where large, densely packed inflammatory foci were visible throughout a majority of infected lung sections (Figure 2B), and animals began to show disease symptoms including lethargy and decreased responses to stimuli. Thus, despite a lack of disease symptoms and a minimal level of detectable changes in lung pathology, there was an increase in bacterial burden as well as an expansion of neutrophils and other innate immune cell populations in the lung during the pre-inflammatory phase of disease.

### The abundance of neutrophils dictates levels of YopE injection

**Figure 3:**
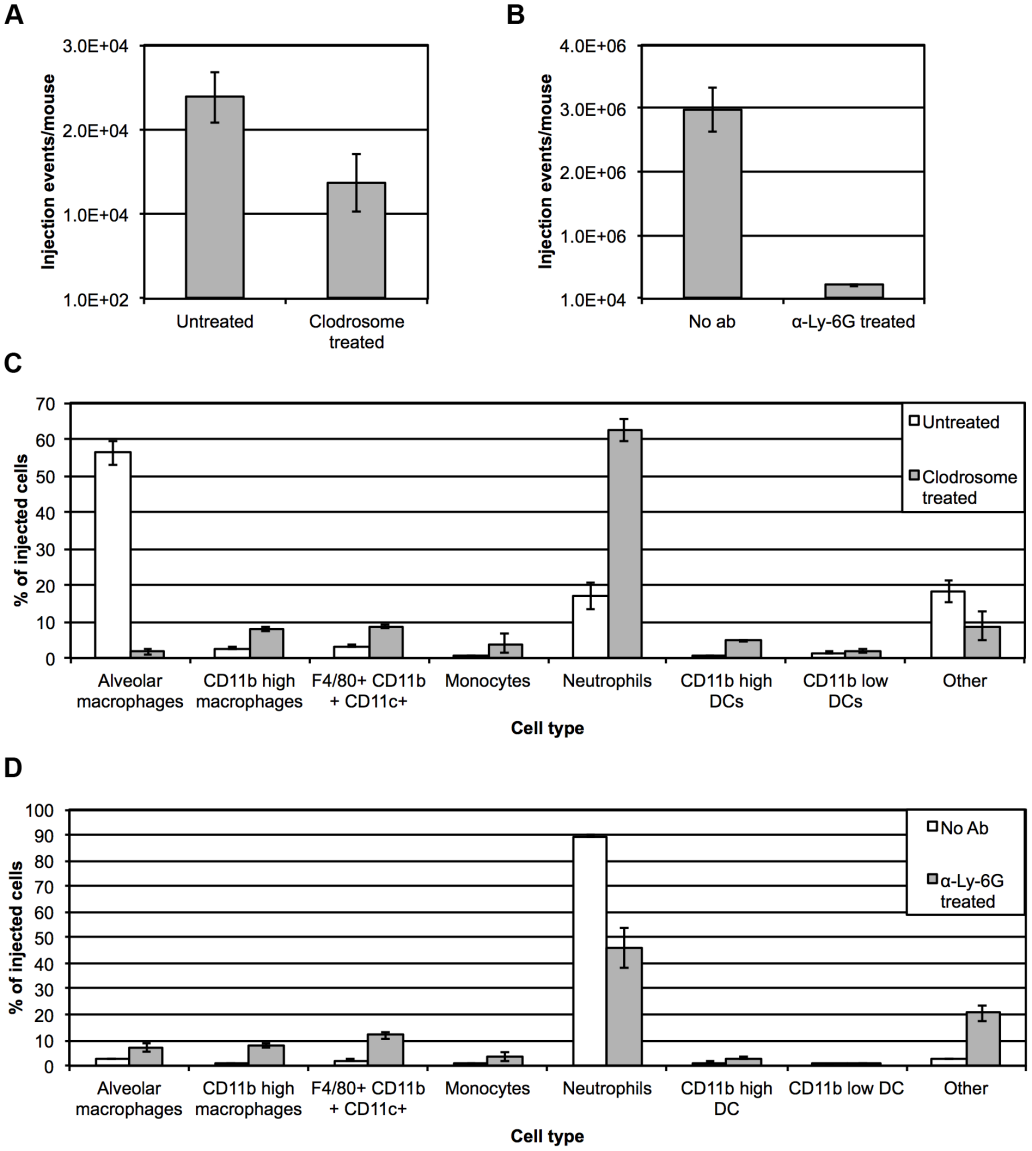
Pre-treatment of mice with clodrosome or neutrophil-depleting antibody α-Ly-6G. (A) Total YopE-TEM injection events 6 hpi in mice inoculated with 10^6^ CFU *Y. pestis* YopE-TEM with and without prior alveolar macrophage depletion with clodrosome. (B) Total YopE-TEM injection events 24 hpi in *Y. pestis*-infected mice with and without pre-treatment with neutrophil-depleting antibody. (C) Identity of injected cells 6 hpi in mice with and without prior clodrosome treatment. (D) Identity of injected cells 24 hpi with and without pre-treatment with neutrophil-depleting antibody. Data are representative of at least three independent experiments with three to five mice at each time point. Error bars represent SEM for all graphs.

At 6 hpi, alveolar macrophages represented the primary cell population targeted by *Y. pestis* type III secretion (Figure 1E). We next sought to evaluate the effect of alveolar macrophage depletion on YopE injection. Clodrosome is a multilamellar liposome suspension encapsulating the drug clodronate (Encapsulum). Liposomal clodronate has been used previously to deplete alveolar macrophages by distillation via the intranasal route [14], [15], [16]. In order to evaluate *Y. pestis* early interactions with alveolar macrophages, mice were treated with clodrosome for two days prior to inoculation with *Y. pestis* YopE-TEM. Intranasal administration of clodrosome resulted in a roughly 92% decrease in alveolar macrophages relative to uninoculated mice (Figure S3A,B). The targeted depletion of alveolar macrophages resulted in a roughly 1.7-fold decrease in total YopE-TEM injection events at 6 hpi (Figure 3A). Analysis of injected cells revealed that with limited numbers of alveolar macrophages available, the primary injected cell type shifted to neutrophils, which represented greater than 60% of injected cells (Figure 3C). This is in contrast to what is seen in untreated mice, where at 6 hpi alveolar macrophages represented roughly 55% of injected cells and neutrophils represented less than 3%.

We sought to determine if the abundance of neutrophils at 24 hpi was the primary factor responsible for an overall increase in injection events. To evaluate levels of YopE-TEM injection in the absence of neutrophils, mice were injected intravenously 24 hours prior to infection with depleting antibody to the lymphocyte antigen 6 complex (Ly-6G). Ly-6G is expressed on the majority of myeloid cells in the bone marrow and peripheral granulocytes, and the use of depleting antibody clone 1A8 has been shown to specifically deplete neutrophils [17], [18]. Treatment with α-Ly-6G antibody resulted in a 99% decrease in F4/80^−^ Ly-6G^+^ cells (Figure S4A,B), and a 90% decrease in total F4/80^−^ Gr-1^+^ cells (data not shown) in the lung while having a minimal impact on other host cell populations, suggesting the efficient and specific depletion of lung neutrophils. The absence of neutrophils resulted in a >10-fold decrease in total injection events at 24 hpi (Figure 3B). Though depletion of neutrophils resulted in an increase in the representation of other cell types in the injected population, neutrophils remained the most prominent host cell target (Figure 3D). This indicates that neutrophils are the preferred host cell target, as their depletion resulted in an overall decrease in total injection events and failed to result in the emergence of an additional single cell type as the primary target. In summary, there is an influx of neutrophils into the lung shortly after inoculation with *Y. pestis*, and as a result, the primary host cell target in the lung shifts from resident alveolar macrophages to neutrophils.

### Depletion of neutrophils attenuates primary pneumonic plague progression in the lung

**Figure 4:**
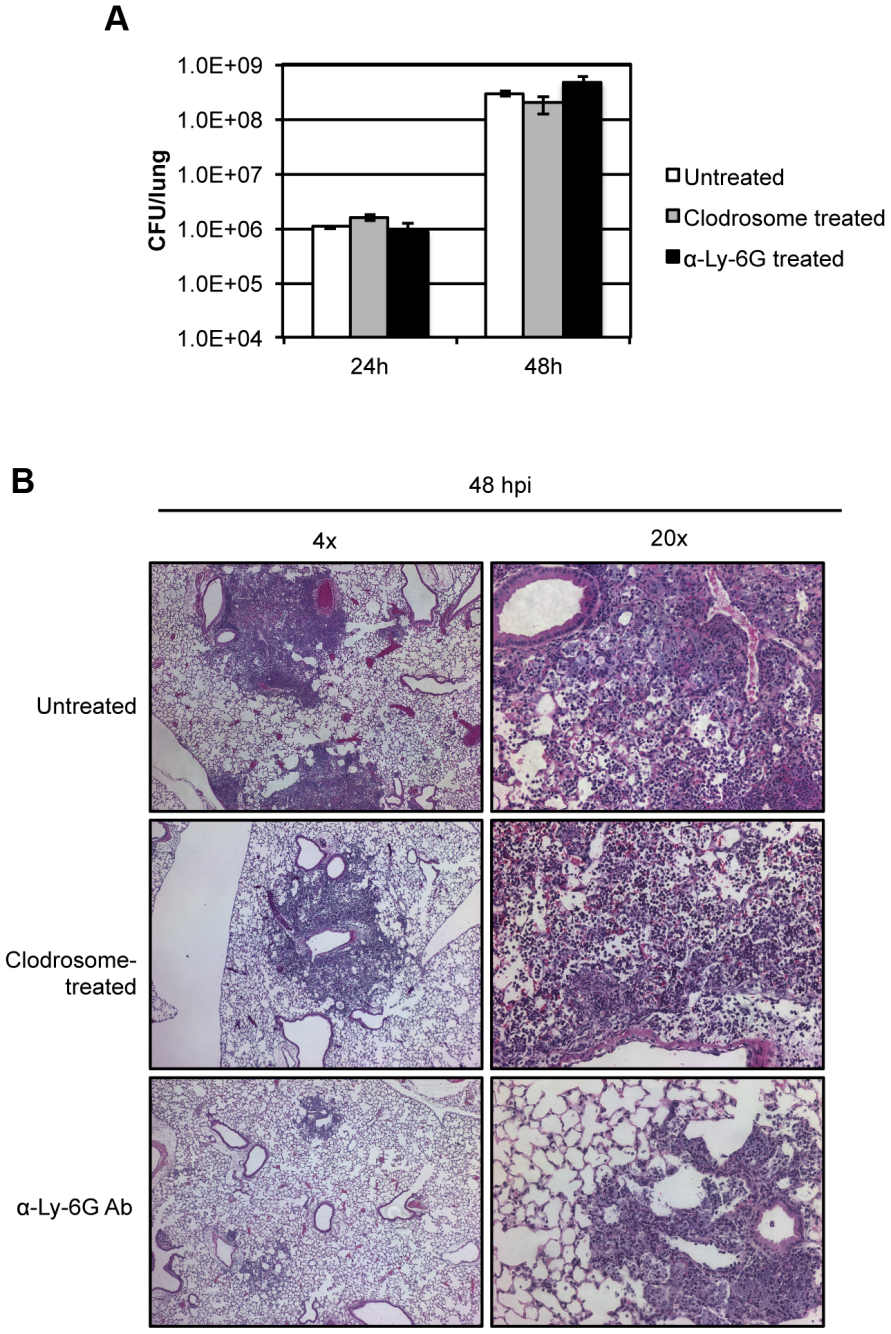
Evaluating the progression of pneumonic plague in mice pre-treated with clodrosome or neutrophil-depleting antibody α-Ly-6G. (A) Lung bacterial burden in mice with and without pre-treatment with clodrosome or neutrophil-depleting antibody and after inoculation with 10^4^ CFU *Y. pestis*; (B) Lung histology 48 hpi in mice inoculated with 10^4^ CFU *Y. pestis* with and without prior treatment with clodrosome or neutrophil-depleting antibody. Bar graph represents experiments repeated at least three times with three to five mice. Error bars represent SEM. Histopathology shows representative images from experiments repeated at least twice.

In order to determine the roles of macrophages and neutrophils in the progression of infection, we evaluated the effects of host cell depletion of each on the progression of primary pneumonic plague. Depletion of alveolar macrophage populations had little effect on bacterial survival in the lung as indicated by bacterial burden analysis at 24 and 48 hpi (Figure 4A). Infected mice pre-treated with clodrosome exhibited a similar time-to-death as untreated infected mice (Figure S5A), and symptoms appeared identical to untreated infected animals at 48 hpi. Histopathology of infected mouse lungs from mice pre-treated with clodrosome revealed the presence of inflammatory foci at 48 hpi that were similar to those in untreated mice (Figures 4B, S5B). These data indicate that depletion of greater than 90% of alveolar macrophages in the lung changed the initial host cell target, but had little effect on progression of primary pneumonic plague.

As neutrophils were the primary host cell target of *Y. pestis* type III secretion (Figure 1E), we hypothesized that depletion of neutrophils would hasten disease progression and enhance bacterial proliferation. Mice pre-treated with α-Ly-6G depleting antibody were infected with the minimum lethal dose of 10^4^ CFU of *Y. pestis* and sacrificed at 48 hpi to determine bacterial burdens in the lung. Depletion of greater than 99% of neutrophil populations had no effect on bacterial burdens the lung at 48 hpi (Figure 4A) and extended mean time-to-death of infected animals by approximately 7 hours, though this difference is not statistically significant (Figure S5A). Surprisingly, mice that were pretreated with α-Ly-6G showed no outward disease symptoms at 48 hpi, whereas untreated infected mice showed increased lethargy and a decreased response to stimuli. Histological evaluation of lungs of infected mice pre-treated with Ly-6G antibody revealed a dramatic decrease in the magnitude and overall area of inflammatory foci compared to that seen in untreated animals (Figures 4B, S5B). Further, whereas the foci found in wild-type infected lungs 48 hpi were densely packed with neutrophils and beginning to lose alveolar structure, lung sections from α-Ly-6G-treated mice demonstrated foci with intact alveolar architecture primarily consisting of bacteria with minimal neutrophil infiltrate (Figure 4B). These data indicate that neutrophils are the primary cell type responsible for the dramatic necrotizing pneumonia attributed to the pro-inflammatory phase of disease. In summary, depletion of neutrophils did not enhance disease, but rather limited progression into the lethal pro-inflammatory phase of pneumonic plague.

## Discussion

The work presented here is the first to identify the initial host cell targets of fully virulent *Y. pestis* during pulmonary infection. Using flow cytometry to monitor injection of a YopE-TEM fusion protein by the T3SS, we showed that *Y. pestis* initially targets CD11c^high^ alveolar macrophages and neutrophils in the lung. Previously, Bosio et al. demonstrated that the attenuated (*pgm*- pCD1-) *Y. pestis* strain A1122 was taken up by a distinct CD11c^+^ CD11b^−^ host population in the lungs following intratracheal inoculation, confirming that *Y. pestis* initially encounters alveolar macrophages early during infection [14]. By 12 hpi there is a dramatic shift in host target cell preference from macrophages to neutrophils that continues to increase by 24 hpi. This corresponds with a massive influx of neutrophils into the lung that is detected as early as 6 hpi. In addition to neutrophils, flow cytometry analysis of infected lungs revealed a 10-fold increase in numbers of CD11b^high^ DCs by 24 hpi. CD11b^high^ DCs are known to accumulate in airways adjacent to alveolar spaces after allergen challenge, and have been implicated in the events that define asthma as well as contributing to CD4^+^ T-cell priming [19], [20]. Infected lungs also saw an overall increase in levels of F4/80^+^CD11b^+^CD11c^+^ cells. These cells exhibited positivity for both interstitial (CD11b^+^) and alveolar (CD11c^+^) macrophage markers and were not targeted by clodrosome in uninfected mice. This population likely consists of a subset of dendritic cells expressing F4/80, activated alveolar macrophages expressing CD11b, and macrophages that have entered the lung in response to bacterial challenge and are induced to express CD11c [21]. Taken together, these results outline a highly dynamic lung environment early during infection where different host innate immune populations are expanding in the lung in an attempt to control *Y. pestis* infection, albeit without much success. Despite the expansion of these cell types, macrophages and neutrophils remained the primary targets of YopE injection.

The *Y. pestis* specificity for injection of macrophages and neutrophils cannot be explained by abundance alone, as the mammalian respiratory tract is comprised of dozens of host cell types with direct access to the airways. We observed minimal injection of CD45^−^CD3^−^ epithelial/endothelial populations at each of the time points examined, despite the fact that this includes alveolar epithelial type I cells which are known to cover roughly 95% of the surface area of the alveoli [22]. Depletion of approximately 92% of alveolar macrophages hastened the shift in target cells seen during normal infection but resulted in only slight increases in levels of injection for other cell types, even though the abundance of these populations was roughly equivalent to that of neutrophils. Conversely, the depletion of roughly 99% of neutrophils in the lung prior to infection resulted in a roughly 10-fold decrease in total injection events. This is similar to what was seen for the enteric pathogen and recent *Y. pestis* ancestor *Y. pseudotuberculosis*, where depletion of neutrophils resulted in a significant overall decrease in total translocation events in Peyer's patches of orogastrically inoculated mice [10]. After treatment with α-Ly-6G antibody, neutrophils remained the primary host cell target. It is known that neutrophils can regenerate rapidly, particularly in the bone marrow, which may partly explain the failed emergence of an additional preferred cell target after depletion of lung neutrophils [23]. The targeting of professional phagocytes, specifically neutrophils, has been seen in other bacterial species and may represent a common virulence mechanism for pathogens harboring a functional T3SS [10], [24], [25]. *P. aeruginosa* has been shown to target macrophages and neutrophils in the lung [24], and *Salmonella enterica* has been shown to target neutrophils in the spleen via type III secretion to promote intracellular survival [25]. This phenomenon also occurs in other organs of the body during *Yersinia* infection. In the first example of *in vivo* detection of Yop translocation via a Yop-β-lactamase fusion, Marketon et al. showed that the attenuated *Y. pestis pgm* ^−^ strain KIM D27 targeted CD11b^+^, CD11c^+^, and Gr-1^+^ cells in the spleen when delivered intravenously, suggesting targeting of macrophages, dendritic cells, and neutrophils [12]. It will be intriguing to compare the injected host cell repertoire of *Y. pestis* delivered via additional routes of infection, including subcutaneous and intradermal, and in other tissues including lymph nodes and in the skin.

As alveolar macrophages and neutrophils are the primary targets of *Y. pestis* type III secretion, we sought to further evaluate their roles during pulmonary infection. Depletion of roughly 92% of alveolar macrophages had little or no effect on bacterial burden in the lung. This indicates that either macrophages are not involved in limiting *Yersinia* survival in the lung, or that *Y. pestis* is able to neutralize the anti-bacterial effects of sentinel alveolar macrophages, presumably as a result of antiphagocytic/anti-inflammatory factors including the Yops, F1 capsular protein, and pH 6 antigen [2]. Also, depletion of alveolar macrophages did not significantly alter progression into the pro-inflammatory phase of disease. Infected animals demonstrated typical disease symptoms as well as the formation of inflammatory foci in infected lungs. Thus, the absence of greater than 90% of alveolar macrophages did not inhibit early host signaling that leads to the massive influx of neutrophils into the lung during pulmonary *Y. pestis* infection. This indicates that either the remaining number of alveolar macrophages is sufficient, or an additional cell type is responsible for this early host response.

Neutrophils have been shown to play a fundamental role in protecting the host during pulmonary infection with a number of organisms, including *Streptococcus pneumoniae*, *Klebsiella pneumoniae*, and *Legionella pneumophila* [26], [27], [28], [29]. We hypothesized that deleting these key mediators of innate immunity would significantly exacerbate disease progression. Surprisingly, depletion of neutrophils had little to no effect on bacterial burden in the lung. This is in contrast to what has been reported previously, as Laws et al. reported a modest increase in bacterial titers in lungs of infected mice early after neutrophil depletion [30]. Differences in mouse lines and *Y. pestis* strains may have contributed to this disparity, as this group utilized a strain of *Y. pestis* that has poor fitness in the lung, as demonstrated by a decrease in bacterial burden by 24 hpi [30]. The unaltered pattern of bacterial proliferation in the lungs of neutrophil-depleted mice indicates that fully virulent *Y. pestis* is highly resistant to the antimicrobial effects of neutrophils that are recruited in response to infection. In support of this, the presence of the *Y. pestis* T3SS has been shown to be important for resisting neutrophil-mediated killing by inhibiting phagocytosis and repressing reactive oxygen species production [31]. Also, gene expression profiling in neutrophils exposed to *Y. pestis* indicated that the T3SS suppresses the activation of a number of genes responsible for pro-inflammatory cytokine output and prolonged cell survival [32]. Surprisingly, infected animals pre-treated with neutrophil-depleting antibody did not demonstrate outward signs of disease at 48 hpi despite significant bacterial burdens, indicating a failure to fully progress into the pro-inflammatory phase of pneumonia. Upon histological examination, the small sporadic foci that were detected in the lungs of these animals were primarily made up of alveolar-residing bacteria and a relatively small number of mononuclear cells and neutrophils. These animals eventually succumbed, not from the typical lung inflammation and pathology of pneumonic plague, but as a result of the unhindered spread of *Y. pestis* to other tissues of the body.

The information gathered in this study supports a model of the pre-inflammatory phase of pneumonic plague where *Y. pestis* initially targets resident alveolar macrophages and then neutrophils to facilitate proliferation in the lung. As neutrophils are unable to sufficiently control *Y. pestis*, their continued chemotaxis results in the formation of massive inflammatory foci and causes severe damage to the alveolar architecture of the lung. Work evaluating the fate of injected macrophages and neutrophils will provide insight into the downstream signaling events that lead to progression into the pro-inflammatory phase of disease.

In summary, the work presented here is the first to identify target host cells and evaluate population dynamics in the lung during primary pneumonic plague. Though much of the previous literature in the field highlights interactions between *Y. pestis* and macrophages, we show that *Y. pestis* primarily targets neutrophils early after inoculation in the lung, presumably to limit host innate immune mechanisms aimed at bacterial killing and clearance. To our surprise, depletion of neutrophils, a key innate immune mechanism for controlling infection during pneumonia, attenuated much of the intense inflammation seen in the lung during the pro-inflammatory phase of disease. Thus, neutrophils appear to be key players in the manifestation of what is ultimately a host-mediated necrotizing pneumonia. Understanding the early delay in innate immune response is crucial to understanding the events that ultimately lead to the progression of this highly lethal syndrome. Further, therapy targeting both bacterial growth as well as the destructive capacity of neutrophils in the lung may be key to expanding the time window during which treatment can be administered during pneumonic plague.

## Materials and Methods

### Ethics statement

The use of live vertebrate animals was performed in accordance with the Public Health Service (PHS) policy on Humane Care and Use of Laboratory Animals, the Amended Animal Welfare Act of 1985, and the regulations of the United States Department of Agriculture (USDA). All animal studies were approved by the University of North Carolina at Chapel Hill Office of Animal Care and Use, protocol #09-057.0.

### Bacterial strains and culture conditions

The fully virulent *Yersinia pestis* strain CO92 was obtained from the U.S. Army, Ft. Detrick, MD. *Y. pestis* strains were grown on brain-heart infusion (BHI) agar (Difco Laboratories) at 26°C for two days. For infections, liquid cultures of *Y. pestis* CO92 were grown in BHI broth for 6–12 h at 26°C. The cultures were then diluted to an OD_620_ of 0.05–0.1 in BHI supplemented with 2.5 mM CaCl_2_ and grown 12–16 h at 37°C with constant shaking. For construction of the YopE-TEM strain, Joan Mecsas (Tufts University) kindly provided us with plasmid pSR47s-E-TEM containing the TEM1 portion of β-lactamase from pBR322 fused to the sequence encoding the promoter plus the first 100 amino acids of YopE [33]. Plasmid pSR47s-E-TEM was transferred into *Y. pestis* CO92 from *Escherichia coli* S17 harboring pSR47-E-TEM via bacterial conjugation in the presence of 10 mM MgSO_4_, followed by growth on BHI agar containing kanamycin (75 µg/mL) and Polymyxin B (25 µg/mL) to select for *Y. pestis* conjugates. Single-copy integration of the plasmid results in two copies of *yopE*: one copy consisting of the native YopE promoter driving the TEM reporter, followed by plasmid sequence and the intact YopE promoter present on the plasmid driving a wild-type YopE open reading frame.

### Animals and animal infections

Six to eight-week-old female C57BL/6J mice were obtained from Jackson Laboratories. Mice were provided with food and water ad libitum and maintained at 25°C and 15% humidity with alternating 12 h periods of light and dark. For animal infections, groups of four to five mice were lightly anesthetized with ketamine/xylazine and inoculated intranasally with the lethal dose of 10^4^ colony forming units (CFU) suspended in 20 µL PBS. A dose of 10^6^ CFUs was necessary for analysis of YopE injection in order to assure sufficient detection in vivo at early time points. Numbers of CFUs were determined by plating serial dilutions of the inoculum on BHI. Moribund animals were euthanized with an overdose of sodium pentobarbital. For determination of bacterial burden, lungs were removed at the indicated times and homogenized in 1 mL PBS using an Omni Tissue Tearer. Serial dilutions of each organ homogenate were plated on BHI agar and reported as CFU/lung.

### Depletion of innate immune cell populations

For depletion of neutrophils, mice were inoculated via tail vein injection with 20 µg LEAF purified anti-mouse Ly-6G Ab clone 1A8 (Biolegend) diluted in 100 µl PBS 24 hours prior to infection. For depletion of alveolar macrophage populations, mice were anaesthetized with katamine/xylazine and clodrosome (Encapsula NanoSciences) or manufacturer-provided control liposomes were delivered intranasally in five successive 15-µL doses. This process was repeated 24 h later, and mice were typically infected 24 hours after the second treatment. For both neutrophil and alveolar macrophage depletion, mice were sacrificed at various times post-inoculation, and lung cells were analyzed by flow cytometry (see below) to evaluate the presence/absence of the appropriate cell types.

### Histopathology

Groups of three mice were inoculated intranasally as described above, and lungs were inflated with 1 mL of 10% neutral buffered formalin via tracheal cannulation, then removed and incubated in 10% formalin for a minimum of 12 h. Lungs were washed once in PBS for 2 hours, immersed in 70% EtOH, and embedded in paraffin. Three five-micrometer sections 200 µm apart per lung were stained with hematoxylin/eosin for examination.

To evaluate the average area of inflammation, images were taken at 4× magnification from inflamed mouse lung sections (three sections per mouse) from at least six mice per treatment condition (untreated, antibody-treated, or clodrosome-treated) from two independent experiments. A total of 30 sections per each treatment condition were examined and inflammatory lesions were defined and area was quantified using ImageJ software [34].

### Generating single-cell suspensions from lungs

For generating a lung single-cell suspension for flow cytometry, mice were euthanized with an overdose of sodium pentobarbital, and the hepatic portal vein was cut and bled. The tracheas were cannulated with a 22G catheter, and lungs were inflated with 1 mL (5 U/mL) Dispase I (BD Biosciences) [13]. Tracheas were tied off with silk thread, and lungs were removed and incubated in Dispase I (5 U/mL) at room temperature for 25 minutes. Lungs were then placed in a petri plate with PBS-DNase I (100 µg/mL, Sigma), and tissue was teased apart using forceps. At this point the trachea and surrounding connective tissue were removed. When most of the tissue was disrupted into suspension, cells were gently swirled for 1 minute, followed by passage through a 100-µm nylon cell strainer (BD Falcon). Filtered suspensions were spun at 500×g for 5 minutes at 4°C, then resuspended in 1 mL red blood cell lysis solution (0.15 M NH_4_Cl, 10 mM KHCO_3_, 0.1 mM EDTA). After incubation at room temperature for 2 minutes, 9 mL PBS was added to neutralize osmolarity, and cells were pelleted as above for staining.

### Staining of lung cell suspensions for flow cytometry

After red blood cell lysis, cell suspensions from each animal were resuspended in 100 µL of 2.4G2 hybridoma supernatant and incubated at room temperature for 20 minutes to block macrophage Fc receptors. Cell suspensions were then pelleted at 500×g for five minutes at 4°C. Cells were resuspended and incubated for 30 minutes at 4°C with the following fluorescent labeled antibodies in flow cytometry buffer (2% fetal bovine serum in PBS) for staining of cell surface markers (1∶100 dilutions): CD11b-phyoerythrin (Clone M1/70.15; Invitrogen), CD11c-phycoerythrin-Texas Red (Clone N418; Invitrogen), Ly6G-phycoerythrin-Cy7 (Clone 1A8; BD Bioscience), LIVE/DEAD fixable aqua dead cell stain (Clone L34957; Invitrogen), and F4/80-allophycocyanin (Clone BM8; Biolegend). Stained cells were analyzed based on fluorescence staining patterns to identify alveolar macrophages (F4/80^+^CD11b^mid/low^CD11c^high^), CD11b^high^ interstitial/exudate macrophages (F4/80^+^CD11b^high^CD11c^low/mid^), F4/80^+^CD11b^high^ CD11c^high^ macrophages, monocytes (F4/80^−^CD11b^high^CD11c^low^Ly-6G^−^), CD11b^high^ and CD11b^low^ dendritic cells (F4/80^−^CD11c^high^CD11b^high or low^), and neutrophils (F4/80^−^CD11c^low^CD11b^high^Ly-6G^+^) [11], [12], [13], [14], [23]. In the absence of CCF2-AM addition, 500 µL PBS was added to samples, followed by centrifugation at 500×g at 4°C and fixation as described below. For addition of CCF2-AM substrate after antibody staining, we prepared 6× CCF2-AM in loading solution (Invitrogen) per manufacturer's instructions, which was diluted into each sample to a final 1× concentration. After incubation at room temperature for 30 minutes, the samples were diluted with PBS and centrifuged at 500×g at 4°C. Cells were then resuspended in 2% formalin and incubated at room temperature for 15 minutes for fixation, followed by centrifugation and resuspension in PBS with gentamicin for removal from the Biosafety Level 3 laboratory.

## Supporting Information

Figure S1 ***Y. pestis*** **primarily targets CD3^−^CD45^+^ innate immune cells for injection of YopE-TEM.** To discriminate between epithelial/endothelial (CD3^−^CD45^−^), innate immune leukocyte (CD3^−^CD45^+^), and lymphocyte (CD3^+^CD45^+^) cell populations, lungs were harvested from groups of mice (n = 3) inoculated with 10^6^ CFU *Y. pestis* YopE-TEM, and stained with antibodies against CD3 and CD45. Samples were analyzed by flow cytometry to discern (A) percentage of each population represented in total recovered cells, (B) the percentage of injected cells represented by each cell type, and (C) the percentage of each cell population demonstrating blue fluorescence, and therefore injection of YopE-TEM. Data is representative of at least two independent experiments. Error bars represent SEM.
(TIFF)

Figure S2 **Identifying and evaluating the host cell repertoire during** ***Y. pestis*** **pulmonary infection.** (A) For identification of host-cell types, digested lung homogenates were stained with fluorescent antibodies against F4/80, CD11b, CD11c, and Ly-6G. The gating strategy used to identify various host cell types is shown in representative histograms of data from an uninfected mouse: G1 = interstitial macrophages; G2 = CD11b^+^CD11c^+^ cells; G3 = alveolar macrophages; G4 = CD11b^High^ DCs; G5 = CD11b^Low^ DCs; G6 = monocytes; G7 = neutrophils. (B) The percentage of each of the cell types demonstrating YopE-TEM injection (blue fluorescence) at 6, 12, and 24 hpi was evaluated by flow cytometry; Data are representative of at least three independent experiments with five mice at each time point. Error bars represent SEM.
(TIFF)

Figure S3 **Depletion of alveolar macrophages with clodrosome.** Cells from lungs of mice treated or untreated with clodrosome were harvested and analyzed by flow cytometry 48 h after the first of two treatments to evaluate alveolar macrophage (F4/80^+^CD11b^low/mid^CD11c^high^) populations. (A) Histograms show F4/80^+^ populations from for a single representative mouse (B) Bar graphs show quantitation of host cell types in PBS (mock)-treated animals or animals treated with clodrosome from a representative of experiments repeated at least twice.
(TIFF)

Figure S4 **Depletion of neutrophils with α-Ly-6G antibody.** Cells from lungs of mice treated with α-Ly-6G antibody, or left untreated, were analyzed for CD11b and Ly-6G expression by flow cytometry 24 h after treatment to evaluate neutrophil (CD11b^+^Ly-6G^+^) populations. (A) Histograms show F4/80^−^CD11c^−^ populations from a single representative mouse; (B) Bar graphs show quantitation of host cell types in PBS (mock)-treated animals or animals treated 24 h prior with α-Ly-6G from a representative of experiments repeated at least twice.
(TIFF)

Figure S5 **Evaluating infected mice treated with α-Ly-6G antibody or Clodrosome.** (A) Mice (n = 10) were left untreated, or pretreated with α-Ly-6G antibody or Clodrosome followed by inoculation with 10^4^ CFU *Y. pestis* CO92. Infected mice were monitored for disease symptoms and were sacrificed when moribund. (B) The lungs of mice inoculated with 10^4^ CFU *Y. pestis* CO92 and treated with α-Ly-6G antibody or clodrosome were harvested at 48 hpi and processed for H and E staining. Lung sections showing inflamed regions were analyzed to calculate the area occupied by inflammatory foci using ImageJ software. Bars represent the area (mm^2^) of inflammation per field in three sections from at least six mice. The mean inflamed area from a total of 30 fields per treatment condition are shown. Asterisks indicate a significant difference in area between sample conditions (* = <.001, ** = <.0001 by two-way ANOVA).
(TIFF)
