# Supplementary figures and images for: Early Host Cell Targets of Yersinia pestis during Primary Pneumonic Plague
Source: PLoS Pathog. 2013 Oct 3;9(10):e1003679. doi: 10.1371/journal.ppat.1003679 (PMC3789773; doi:10.1371/journal.ppat.1003679)

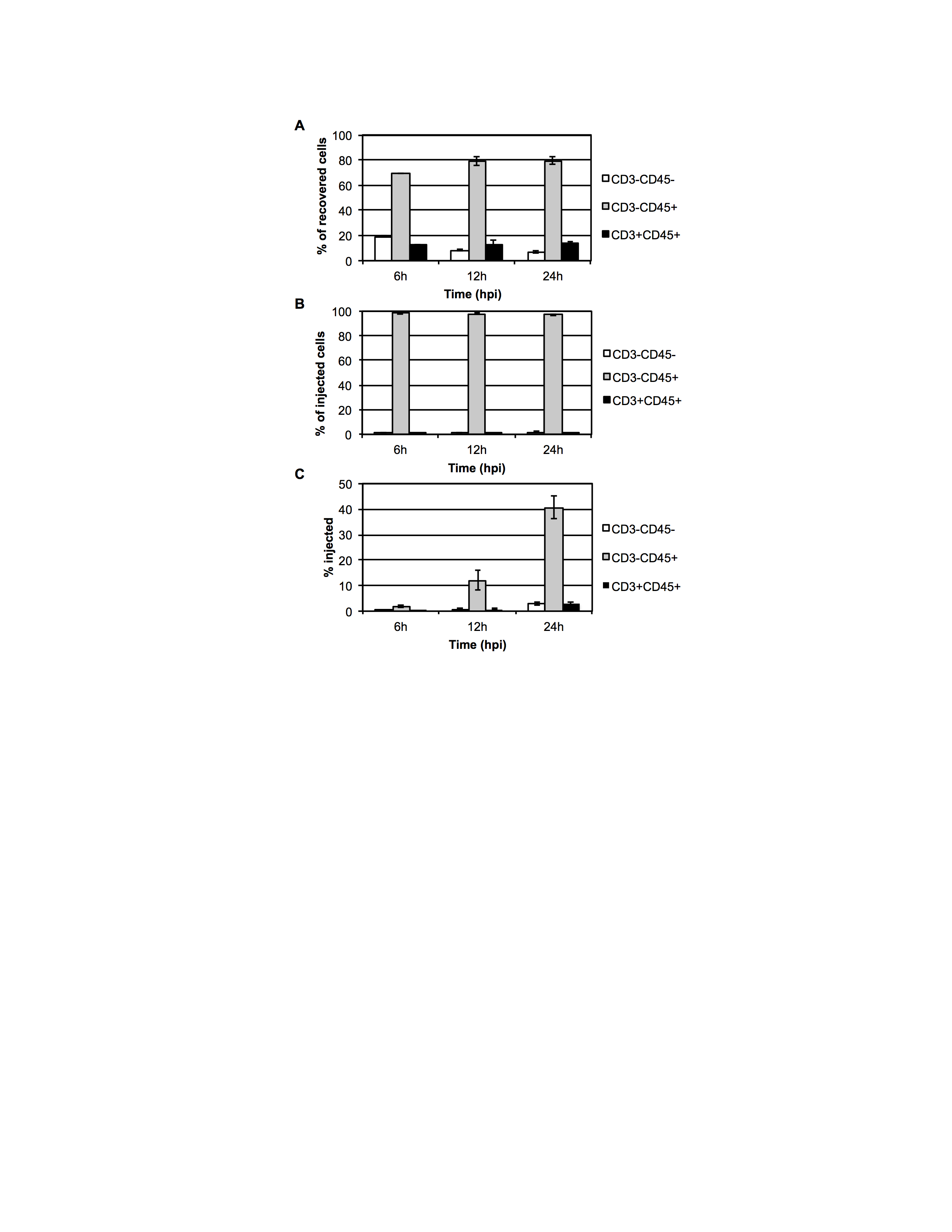

Supplement: Figure S1 — Y. pestis primarily targets CD3−CD45+ innate immune cells for injection of YopE-TEM. To discriminate between epithelial/endothelial (CD3−CD45−), innate immune leukocyte (CD3−CD45+), and lymphocyte (CD3+CD45+) cell populations, lungs were harvested from groups of mice (n = 3) inoculated with 106 CFU Y. pestis YopE-TEM, and stained with antibodies against CD3 and CD45. Samples were analyzed by flow cytometry to discern (A) percentage of each population represented in total recovered cells, (B) the percentage of injected cells represented by each cell type, and (C) the percentage of each cell population demonstrating blue fluorescence, and therefore injection of YopE-TEM. Data is representative of at least two independent experiments. Error bars represent SEM. (TIFF) [file ppat.1003679.s001.tiff]

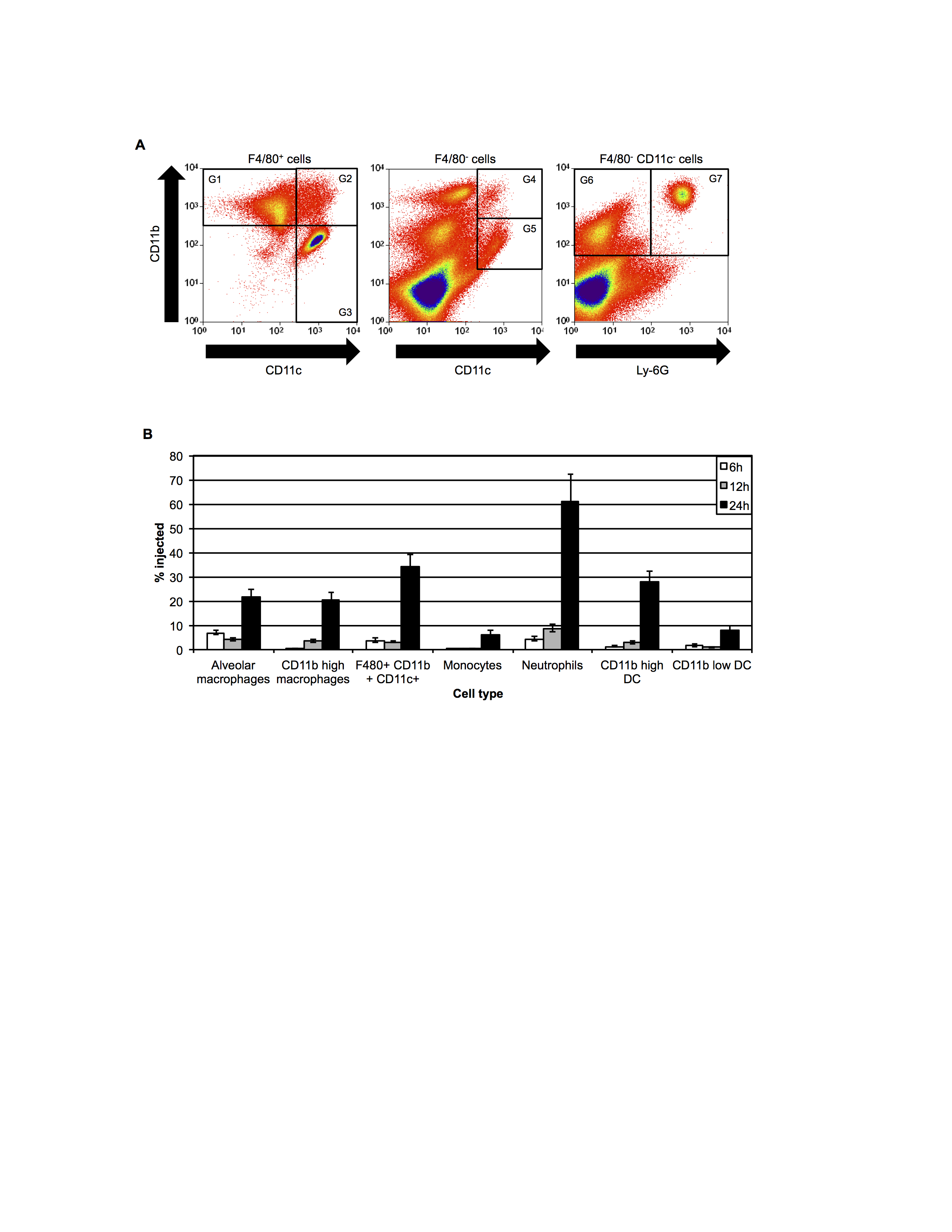

Supplement: Figure S2 — Identifying and evaluating the host cell repertoire during Y. pestis pulmonary infection. (A) For identification of host-cell types, digested lung homogenates were stained with fluorescent antibodies against F4/80, CD11b, CD11c, and Ly-6G. The gating strategy used to identify various host cell types is shown in representative histograms of data from an uninfected mouse: G1 = interstitial macrophages; G2 = CD11b+CD11c+ cells; G3 = alveolar macrophages; G4 = CD11bHigh DCs; G5 = CD11bLow DCs; G6 = monocytes; G7 = neutrophils. (B) The percentage of each of the cell types demonstrating YopE-TEM injection (blue fluorescence) at 6, 12, and 24 hpi was evaluated by flow cytometry; Data are representative of at least three independent experiments with five mice at each time point. Error bars represent SEM. (TIFF) [file ppat.1003679.s002.tiff]

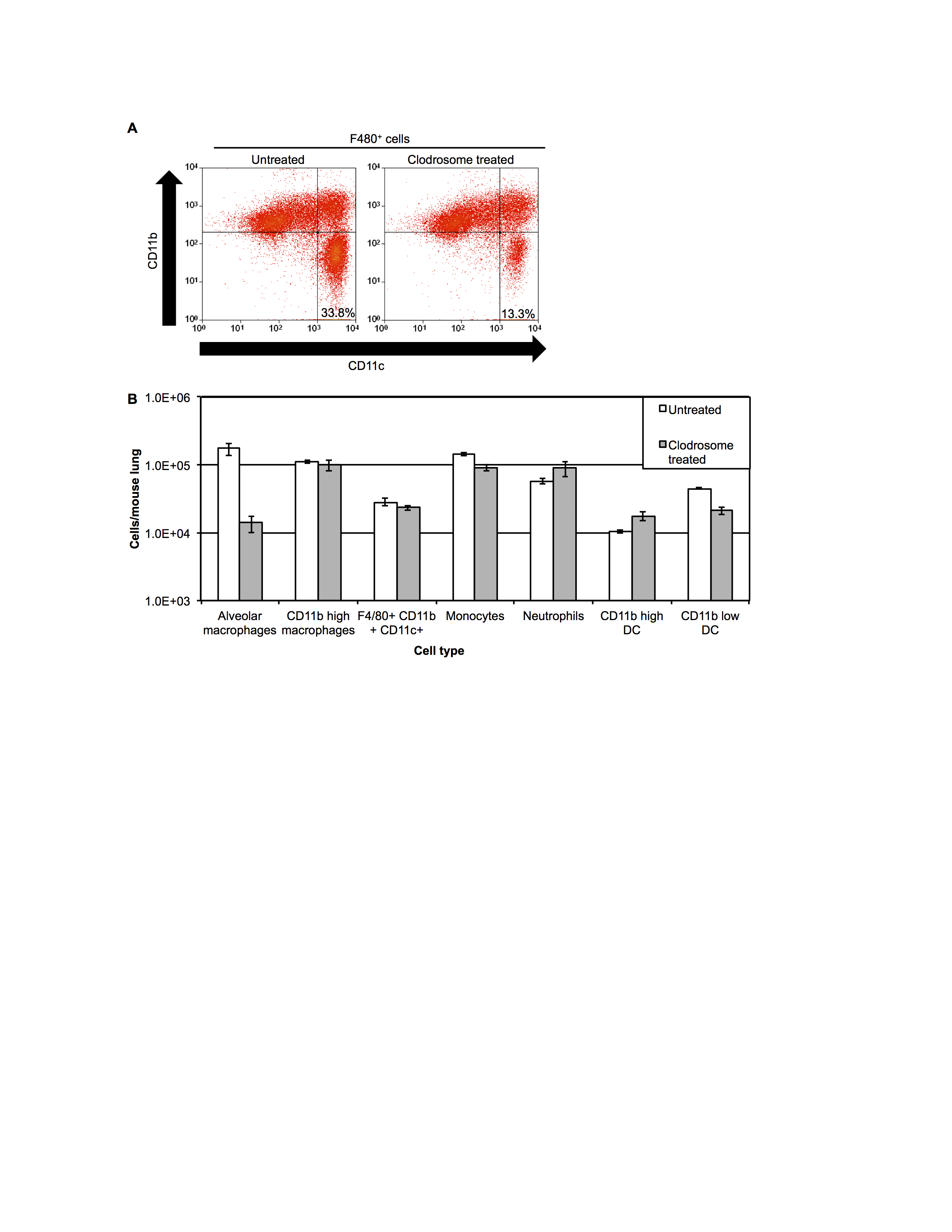

Supplement: Figure S3 — Depletion of alveolar macrophages with clodrosome. Cells from lungs of mice treated or untreated with clodrosome were harvested and analyzed by flow cytometry 48 h after the first of two treatments to evaluate alveolar macrophage (F4/80+CD11blow/midCD11chigh) populations. (A) Histograms show F4/80+ populations from for a single representative mouse (B) Bar graphs show quantitation of host cell types in PBS (mock)-treated animals or animals treated with clodrosome from a representative of experiments repeated at least twice. (TIFF) [file ppat.1003679.s003.tiff]

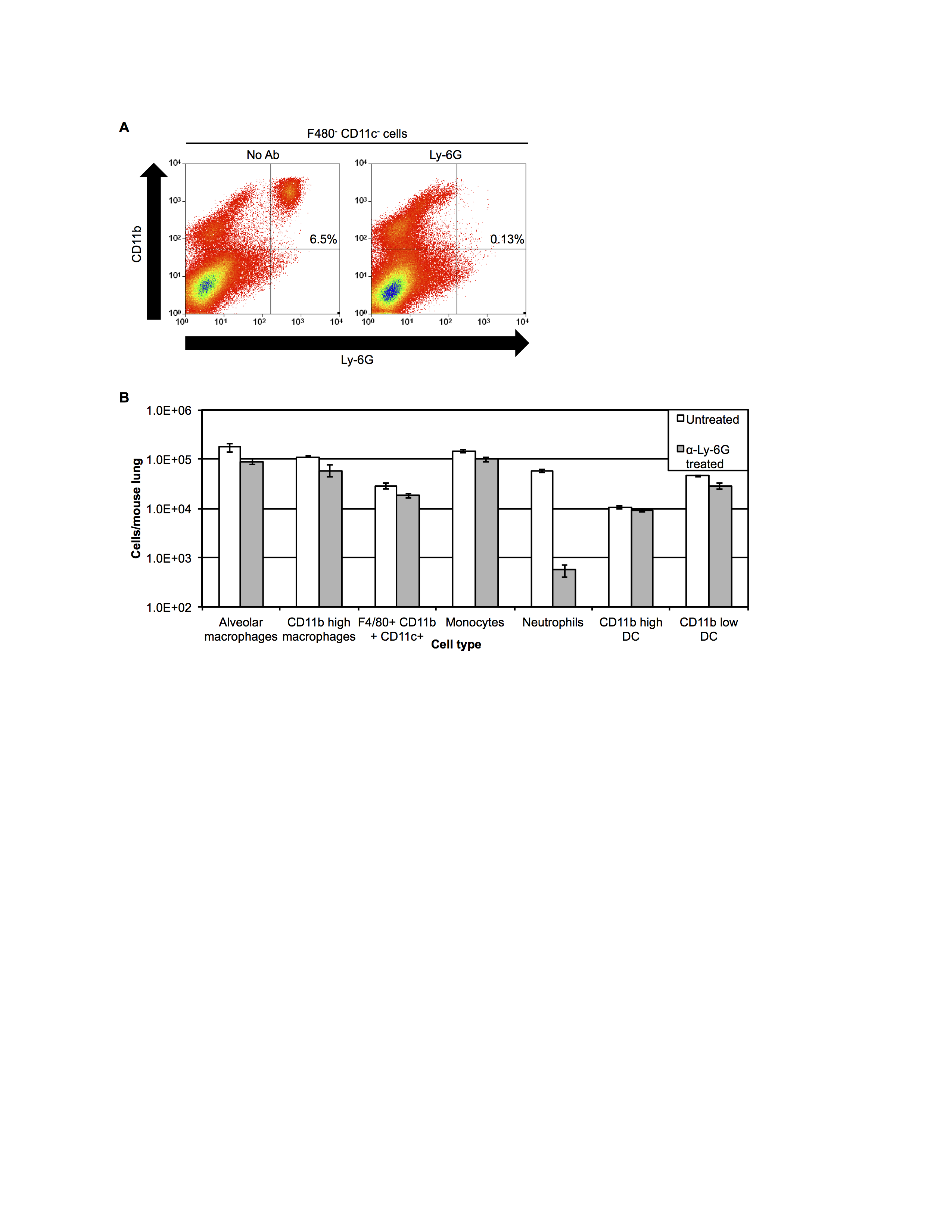

Supplement: Figure S4 — Depletion of neutrophils with α-Ly-6G antibody. Cells from lungs of mice treated with α-Ly-6G antibody, or left untreated, were analyzed for CD11b and Ly-6G expression by flow cytometry 24 h after treatment to evaluate neutrophil (CD11b+Ly-6G+) populations. (A) Histograms show F4/80−CD11c− populations from a single representative mouse; (B) Bar graphs show quantitation of host cell types in PBS (mock)-treated animals or animals treated 24 h prior with α-Ly-6G from a representative of experiments repeated at least twice. (TIFF) [file ppat.1003679.s004.tiff]

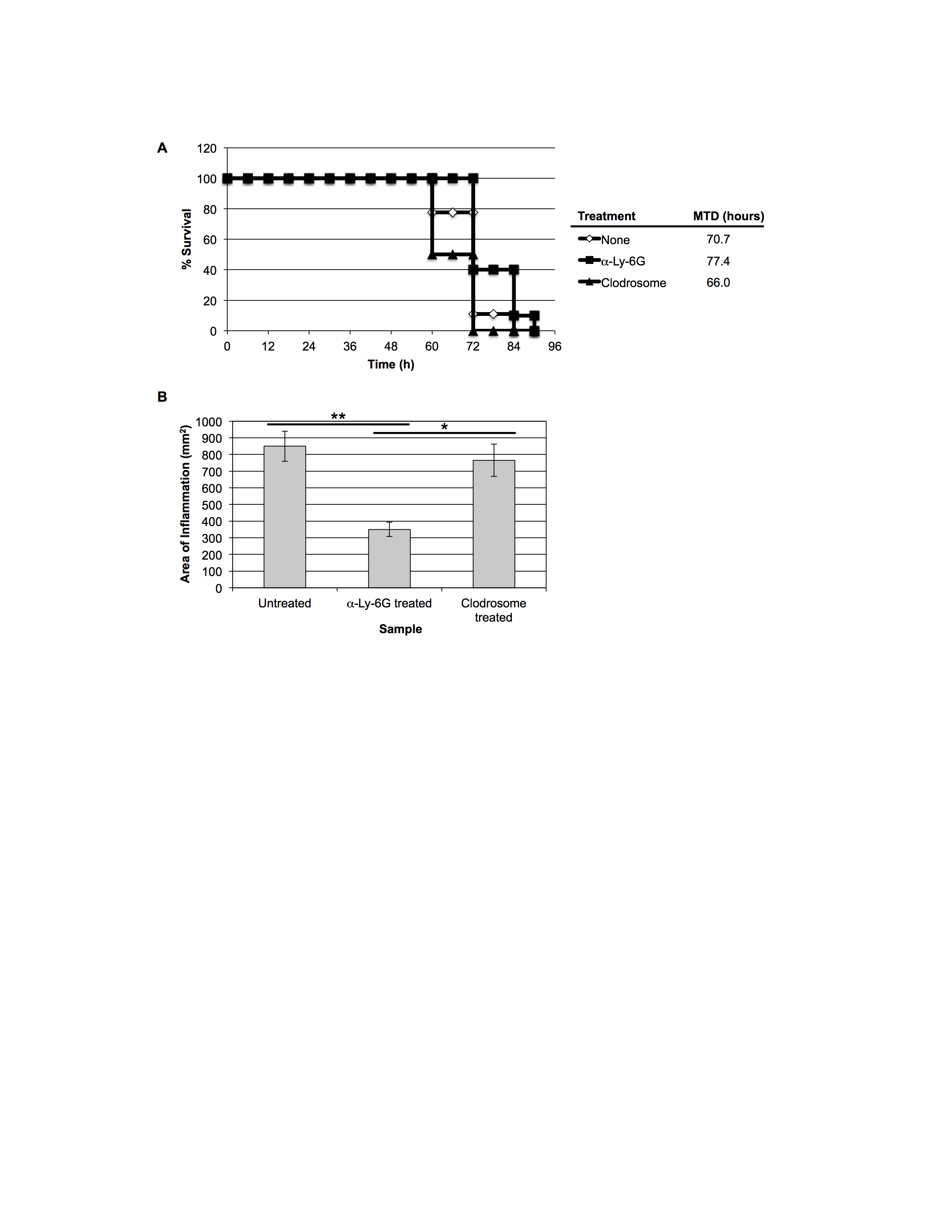

Supplement: Figure S5 — Evaluating infected mice treated with α-Ly-6G antibody or Clodrosome. (A) Mice (n = 10) were left untreated, or pretreated with α-Ly-6G antibody or Clodrosome followed by inoculation with 104 CFU Y. pestis CO92. Infected mice were monitored for disease symptoms and were sacrificed when moribund. (B) The lungs of mice inoculated with 104 CFU Y. pestis CO92 and treated with α-Ly-6G antibody or clodrosome were harvested at 48 hpi and processed for H and E staining. Lung sections showing inflamed regions were analyzed to calculate the area occupied by inflammatory foci using ImageJ software. Bars represent the area (mm2) of inflammation per field in three sections from at least six mice. The mean inflamed area from a total of 30 fields per treatment condition are shown. Asterisks indicate a significant difference in area between sample conditions (* = <.001, ** = <.0001 by two-way ANOVA). (TIFF) [file ppat.1003679.s005.tiff]
